# THE ASSOCIATION BETWEEN ORAL HEALTH/HYGIENE AND COGNITIVE IMPAIRMENT AMONG MULTIETHNIC OLDER ADULTS IN WEST CHINA

**DOI:** 10.1093/geroni/igae098.0089

**Published:** 2024-12-31

**Authors:** Yuqing Xie, Xin Xia, Yun Li, Yanyan Wang, Xin Tian, Yuexia Hu

**Affiliations:** Sichuan university, Chengdu, Sichuan, China (People’s Republic); Center of Gerontology and Geriatrics, West China Hospital, Sichuan University/ National Clinical Research Center for Geriatrics, West China Hospital, Sichuan University, Chengdu, Sichuan, China (People’s Republic); Sichuan university, Chendu, Sichuan, China (People’s Republic); West China School of Nursing, Sichuan University, Chengdu, Sichuan, China (People’s Republic); Sichuan University, Chengdu, China (People’s Republic); Sichuan university, Chengdu, Sichuan, China (People’s Republic)

## Abstract

The prevalence of cognitive impairment increases with age. Meanwhile, older adults with cognitive impairment typically have deteriorating oral health and poor oral hygiene. However, the distribution of oral health and cognitive impairment varies among different ethnic groups. The aim of this cross-sectional, multi-center study was to investigate the association between oral health and hygiene and cognitive impairment in a sample of multi-ethnic older adults aged 50 years and older from four provinces in western China (N=6529, mean age 62.4 ±8.3 years). Oral health and hygiene were assessed by specialized oral tests and a self-made questionnaire. Cognitive status was assessed using the Chinese version of the Short Portable Mental Status Questionnaire (SPMSQ). Three multiple regression models were used to examine the association between oral health and hygiene and cognitive impairment, adjusting for relevant variables. Statistical analysis showed that the prevalence of cognitive impairment was 15.4%, with the Yi group having the highest prevalence (28.9%), followed by Tibetans and others. Poorer self-rated oral health, fewer residual teeth, less frequency of using toothbrush and toothpaste, and rare dental visits increased the risk of cognitive impairment. The SPMSQ scores and correlations between oral health and hygiene and cognitive impairment were heterogeneous across the multi-ethnic groups. These findings highlight that declining oral health and inadequate oral hygiene may increase the risk of cognitive impairment. The potential value of this study is to illustrate that implementing and promoting oral health and hygiene is essential to prevent cognitive impairment in multi-ethnic older populations.

